# On Constipation

**Published:** 1877-07-20

**Authors:** Wm. H. Thompson

**Affiliations:** New York City; Professor of Therapeutics in the Medical Department of the University of the City of New York


### ON CONSTIPATION.

### CLINICAL LECTURE DELIVERED IN BELLEVUE HOSPITAL.

By WM. H. THOMPSON, M. D.,
Professor of Therapeutics in the Medical Department of the University of the City of New York.

GENTLEMEN:—I will direct your attention to-day to the treatment of constipation as found among males as commonly, perhaps, as among females. The constipation generally complained of in the male sex I divide into that due to deficient action of the small intestine, and into that due to deficient action on the part of some portion of the large intestine.

Deficient action on the part of the small intestine is due to two causes:

1. Deficient secretion;
2. Want of innervation, or want of muscular action.

Constipation dependent upon deficient secretion is quite distinct from that caused by want of muscular action, and yet you will have many cases in which both causes are operating.

Deficient secretion in the small intestine may be caused by some disturbance of the liver. Constipation, therefore, may date from the time when the patient suffered from some severe form of fever in which the liver was prominently involved, such as the bilious remittent; or it may follow an attack of tropical diarrhœa, which is almost invariably accompanied by marked hepatic disturbance. In such cases the patient does not have an extraordinary fecal accumulation and impaction, but there is, instead, a sluggish action of the bowels, and they are usually obliged to take medicine to bring about a movement once in four or five days; and when it does occur, the evacuation is moderate in amount, and quite dry. This kind of constipation is quite common in the Southern States, as a sequence of the diarrhœa which prevails in that latitude; and it is also frequently seen in the Northern States as the result of malarial poisoning.

The symptoms are extremely negative, except the constipation. The one which, perhaps, gives the patient most discomfort, is a tendency to a dull, indefinite headache. In a majority of cases this is located in the posterior part of the head, is rather an uncomfortable sensation than a real pain, and is best relieved by something which promotes a free discharge of bile. The tongue usually is small, not large and flabby, generally a little reddened along the edges and tip, and the secretions of the mouth are commonly viscid. The condition of the mouth is an indication of the condition present along the entire alimentary canal. We have, therefore, evidence of the presence of only a moderate amount of secretion in the intestinal tube, and our treatment should be regulated accordingly.

If, for the relief of this condition, you administer mild cathartics, the condition of the case will be aggravated, because the temporary stimulus afforded by them, however mild, is immediately overcome by the tendency to deficient secretion. Active purgation produces a much more injurious effect than mild laxatives. If you resort to the use of medicines which have been recommended to stimulate nerve action, you will not obtain much benefit. What you wish to have present in the intestine, is a small increase of lubricating substance, as it were, and, to that end, I have found altogether the best results have been obtained by causing the patient to take a great deal more water than is his usual custom. Let him take, on rising in the morning, two tumblerfuls of Croton or other drinking-water. As a rule, those who drink considerable water are not troubled with constipation. You can insure the laxative action of the water by the addition of some mild saline, like the carbonate of soda, or even common salt, and the reason why such an effect is produced is this: the mixture formed by the union of some saline with water, does not readily pass through the mucous membrane, and so into the general system. The theory now generally accepted with regard to the action of salines is that they are not absorbed, and that they prevent the water with which they are combined from being absorbed; hence the water, by exciting the peristaltic action of the bowel, brings about a movement to discharge it, and with that the other contents of the intestinal tube. There is considerable to lend support to this view. You need not, therefore, give large doses of saline cathartics, as a half-drachm of the sulphate of magnesia, dissolved in a pint of water, commonly operates very nicely.

There is another curious fact which may here be mentioned, namely: the addition of small doses of quinine to salines increases their power of acting upon the intestine. For example:

R. Magnesia sulphas...........1 dr.
Quin sulph.................1 gr.

mixed and taken in a tumbler of water every morning rarely fails to produce all the laxative effect required, in every form of deficient secretion from the bowels; for instance, in the constipation following fever, when you desire to obtain a free alvine evacuation.

It is well for you to tell the patients that they will not, perhaps, see much effect for one or two weeks, but if they can be induced to persist in the daily use of large quantities of water, a great deal of benefit will almost certainly follow. There is a supposition on the part of the laity that certain fruits are laxative, and that is probably true to a limited extent. *Oranges* may be eaten with benefit, but it usually requires ten or twelve to overcome an obstinate constipation, a fact which renders the remedy quite impracticable in this climate. In the warmer climates, however, the worst forms of constipation which appear can be overcome by oranges alone, and the more juicy they are the better, from the fact that the citric acid which they contain has a tendency to produce a catarrh of the intestine if taken in excess. *Figs* are a rather dangerous laxative, for they may obstruct the intestines; there is not much danger, however, in this direction, if taken with a large quantity of water. It will be found necessary to use about double the amount of water with figs that will be required with any other laxative fruit. The fruits of this climate are very uncertain in their action; the action of apples is very good, but a great many persons are unable to take them in sufficient quantity to produce any effect upon the bowels, although they may at the same time take a large quantity of water. All along you will find that water is one of the most important agents to be employed for overcoming deficient secretion in the intestine, attending constipation. If *flatulence*, resulting from decomposition of the intestinal secretion, accompanies the constipation, you may have recourse to the following pill:

R. Assafœtida.................gr. iv.

Saponis....................gr. ix.

M.

To this may be added nux vomica, if there is evidence of *deficient innervation* in the intestine.

How are you to judge that the leading element in the case is deficient innervation? I am now speaking with more especial reference to the small intestine. As a rule you may say with safety that deficient innervation is an accompaniment of the constipation that troubles persons with sedentary habits of life.

In this class of cases nux vomica has proven itself a very efficient remedy, and it may be administered in connection with any drug you may wish to use. It will increase the efficacy of small doses of the resinous cathartics, which are irritant and stimulant; hence small doses of rhubarb with nux vomica and soap, may be given in the form of a pill with much more benefit than when administered separately,

The application of the faradic current, one pole of the battery placed over the spine, and the other passed up and down over the abdominal walls, will, in many cases, be found beneficial.

What is known as the health-lift will prove advantageous in certain cases, and the reason is that it brings into action all the abdominal muscles, especially the recti, and that action is brought to bear directly upon the sluggish intestines. When any lesion of the bowels is present, the health-lift cannot be employed.

CONSTIPATION DEPENDENT UPON CERTAIN CONDITIONS PRESENT IN THE LARGE INTESTINES.

We now come to the large intestine, and here we find that constipation depends upon nearly the same conditions as were found present in the small intestines. That is, we have constipation dependent upon deficiency of action, and that it in turn may depend upon deficient secretion or deficient innervation, but is far more commonly dependent upon the latter. Here the patient may be troubled with large fecal accumulations, and that condition may depend upon deficient nerve power on the part of the colon, or the deficient innervation may be confined to the rectum.

One of the worst forms of constipation may occur, dependent upon no other condition than that which is present in the rectum alone, and unless the physician is upon the alert, the result may be the development of a rectal abscess.

When this condition is present, the patients have but little knowledge that they should have a movement from the bowels, and whenever the sensation is developed, they have little or no power to expel the fecal accumulation. When such symptoms are present, it is a pretty certain indication that they depend upon deficient innervation of the rectum, and, unless that condition is overcome, serious consequences may follow. One of the most common causes of this condition is a chronic inflammation set up about hemorrhoids. Prolonged inflammation of any part, especially, however, about the mucous membrane, produces deficient innervation, and then follows a relaxed condition, and with this deficient innervation we are, therefore, very liable to have prolapsus of the rectum.

These patients are peculiar in one respect, namely: they are very generally low-spirited. It sometimes happens that insanity is developed by such a diseased condition of the rectum, and is relieved when the rectal trouble is removed.

With regard to treatment, the first indication is to keep the rectum empty. When fecal accumulations are present, the most efficient and convenient method of removing them is by means of enemata; but just here I wish to say a few words of caution with reference to resorting to that measure. You should never prescribe enemata as a regular treatment, for if the patient gets into the way of emptying the bowels daily in health by enemata, they can never dispense with their use. If you recommend that the patient use the syringe every morning for the purpose of evacuating the bowels, and it is continued regularly for six weeks, he has gone considerably far towards making it a necessity during the remainder of his life. Do not abuse the measure if you can possibly avoid doing so. It will probably be necessary to use this means for removing accumulations which happen to be present, but when they are thoroughly cleared out you should at once resort to other measures for restoring lost innervation to the bowel, and one of the very best of these is the local use of strychnia. It is an exceedingly valuable specific in these cases.

It will frequently succeed in curing the worst forms of prolapsus of the rectum, as well as that condition in which there is simple debility with hypertrophy of the mucous membrane. The manner in which you can carry long-standing cases of prolapsus of the rectum by means of injections of strychnia into the submucous tissue itself is sometimes wonderful. If necessary, you can draw a fold of the mucous membrane down, and then insert the injection.

I have relied upon this agent almost exclusively in the treatment of this class of cases, whether the real cause was hypertrophy of the mucous membrane from long-standing hemorrhoids, or there was a simple deficiency of power in the rectum to expel its contents.

